# Supplementary material for: Social harmony at work: A sharedness index linking team atmosphere to individual well-being in a Japanese company
Source: PLoS One. 2025 Dec 29;20(12):e0336368. doi: 10.1371/journal.pone.0336368 (PMC12747401; doi:10.1371/journal.pone.0336368)
Supplement: S1 Table — (DOCX) [file pone.0336368.s001.docx]

**S1 Table. All items on the Interdependent Happiness Scale (Hitokoto & Uchida, 2014).**

Since the questionnaire was conducted in Japanese, the English and Japanese versions are presented side by side.

| English items | Japanese items |
| --- | --- |
| I believe that I and those around me are happy. | 自分だけでなく、身近なまわりの人も楽しい気持ちでいると思う。 |
| I feel that I am being positively evaluated by others around me. | 周りの人に認められていると感じる。 |
| I make significant others happy. | 大切な人を幸せにしていると思う。 |
| Although it is quite average, I live a stable life. | 平凡だが安定した日々を過ごしている。 |
| I do not have any major concerns or anxieties. | 大きな悩み事はない。 |
| I can do what I want without causing problems for other people. | 人に迷惑をかけずに自分のやりたいことができている。 |
| I believe that my life is just as happy as that of others around me. | まわりの人たちと同じくらい幸せだと思う。 |
| I believe I have achieved the same standard of living as those around me. | まわりの人並みの生活は手に入れている自信がある。 |
| I generally believe that things are going well for me in its own way as they are for others around me. | まわりの人たちと同じくらい、それなりにうまくいっている。 |
