# Supplementary material for: Social harmony at work: A sharedness index linking team atmosphere to individual well-being in a Japanese company
Source: PLoS One. 2025 Dec 29;20(12):e0336368. doi: 10.1371/journal.pone.0336368 (PMC12747401; doi:10.1371/journal.pone.0336368)
Supplement: S2 Algorithm — (DOCX) [file pone.0336368.s002.docx]

**S2 Algorithm. Computation of TSI for a team by week.**

**Input**
Weekly diaries of team t in week w; pre-trained Word2Vec model M (Japanese Wikipedia Entity Vectors, 300d)

**Output**
TSIₜ,₍w₎

**Algorithm**

1. For each member i in team t, concatenate all diary entries within week w to form a document dᵢ.
2. Tokenize dᵢ to obtain a token sequence xᵢ (Japanese morphological segmentation).
3. Collect X = {xᵢ} for all members with data in the team by week and require |X| ≥ 2. Otherwise exclude the team by week.
4. Build a gensim WmdSimilarity index I over X using model M (num_best = |X|).
5. Initialize S = 0 and C = 0.
6. For each unordered member pair (i, j) with i < j:
   • Query I with xᵢ to obtain similarities to all texts in X.
   • Retrieve the similarity for member j and update S = S + sim(xᵢ, xⱼ); then update C = C + 1.
7. Return TSIₜ,₍w₎ = S / C as the mean pairwise similarity across all member pairs.

**Implementation details**

We used gensim’s WmdSimilarity (v3.8.3) with the Japanese Wikipedia Entity Vectors Word2Vec model (2019-05-20). Tokens were produced with MeCab and joined with single spaces. Repeated whitespace was collapsed to a single space. Tokens absent from the embedding vocabulary were ignored by WMD. Member-by-week documents with no in-vocabulary tokens were dropped. Team-by-week units with fewer than two contributors were excluded.
