# Supplementary material for: Social harmony at work: A sharedness index linking team atmosphere to individual well-being in a Japanese company
Source: PLoS One. 2025 Dec 29;20(12):e0336368. doi: 10.1371/journal.pone.0336368 (PMC12747401; doi:10.1371/journal.pone.0336368)
Supplement: S3 Table — (DOCX) [file pone.0336368.s003.docx]

**S3 Table. Team-stratified correlations**

| Team | r (SSI) | p (SSI) | N_weeks (SSI) | r (TSI) | p (TSI) | N_weeks (TSI) |
| --- | --- | --- | --- | --- | --- | --- |
| A | -0.750 | 0.086 | 6 | 0.258 | 0.622 | 6 |
| B | — | — | 2 | -0.385 | 0.451 | 6 |
| C | — | — | 3 | 0.681 | 0.319 | 4 |
| G | 0.973 | 0.027 | 4 | 0.365 | 0.635 | 4 |
| I | -0.064 | 0.869 | 9 | -0.027 | 0.946 | 9 |
| J | 0.677 | 0.045 | 9 | 0.067 | 0.865 | 9 |
| K | 0.483 | 0.188 | 9 | -0.046 | 0.906 | 9 |
| L | 0.777 | 0.023 | 8 | -0.540 | 0.167 | 8 |
| M | — | — | 1 | -0.110 | 0.778 | 9 |
| N | 0.544 | 0.130 | 9 | -0.319 | 0.403 | 9 |
| O | 0.400 | 0.286 | 9 | -0.635 | 0.066 | 9 |
| P | -0.075 | 0.873 | 7 | 0.106 | 0.820 | 7 |
| R | 0.256 | 0.507 | 9 | -0.193 | 0.618 | 9 |
| S | -0.488 | 0.182 | 9 | -0.344 | 0.364 | 9 |
| U | — | — | 3 | 0.293 | 0.707 | 4 |
| V | 0.251 | 0.515 | 9 | 0.397 | 0.289 | 9 |
