# Supplementary material for: Social harmony at work: A sharedness index linking team atmosphere to individual well-being in a Japanese company
Source: PLoS One. 2025 Dec 29;20(12):e0336368. doi: 10.1371/journal.pone.0336368 (PMC12747401; doi:10.1371/journal.pone.0336368)
Supplement: S4 Table — (DOCX) [file pone.0336368.s004.docx]

**S4 Table. Weekly correlations**

| Week | r (SSI) | p (SSI) | N (SSI) | r (TSI) | p (TSI) | N (TSI) |
| --- | --- | --- | --- | --- | --- | --- |
| 1 | 0.098 | 0.698 | 18 | 0.242 | 0.319 | 19 |
| 2 | 0.169 | 0.488 | 19 | 0.202 | 0.393 | 20 |
| 3 | 0.729 | 0.002 | 15 | 0.658 | 0.002 | 19 |
| 4 | 0.613 | 0.020 | 14 | 0.465 | 0.070 | 16 |
| 5 | 0.319 | 0.339 | 11 | 0.314 | 0.296 | 13 |
| 6 | 0.050 | 0.877 | 12 | 0.474 | 0.087 | 14 |
| 7 | 0.735 | 0.010 | 11 | 0.546 | 0.066 | 12 |
| 8 | 0.759 | 0.011 | 10 | 0.547 | 0.082 | 11 |
| 9 | 0.755 | 0.030 | 8 | 0.336 | 0.376 | 9 |
