# Supplementary material for: Social harmony at work: A sharedness index linking team atmosphere to individual well-being in a Japanese company
Source: PLoS One. 2025 Dec 29;20(12):e0336368. doi: 10.1371/journal.pone.0336368 (PMC12747401; doi:10.1371/journal.pone.0336368)
Supplement: Attachment — Submitted filename: Response_to_Reviewers.pdf [file pone.0336368.s005.pdf]

We thank you for the careful and constructive evaluation of our manuscript. Guided by your comments, we have substantially revised the paper to improve conceptual framing, structure, methodological transparency, language, figure/table handling, and data availability compliance.

To the editor:

Thank you for your editorial work on the paper. We checked each Common formatting/editorial problem.

Journal Requirements

> Journal Requirements 1. Please ensure that your manuscript meets PLOS ONE's style requirements, including those for file naming.
> The PLOS ONE style templates can be found at
> https://journals.plos.org/plosone/s/file?id=wjVg/PLOSOne_formatting_sample_main_body.pdf
> and
> https://journals.plos.org/plosone/s/file?id=ba62/PLOSOne_formatting_sample_title_authors_affiliations.pdf

Response: Thank you for your comments. We have carefully reviewed the entire manuscript format and made the necessary corrections.

> Journal Requirements 2. Thank you for stating the following financial disclosure:
>
> "This work was supported by JST-Mirai Program Grant Number JPMJMI21J2, Japan."
>
> Please state what role the funders took in the study. If the funders had no role, please state: "The funders had no role in study design, data collection and analysis, decision to publish, or preparation of the manuscript."
>
> If this statement is not correct you must amend it as needed.
>
> Please include this amended Role of Funder statement in your cover letter; we will change the online submission form on your behalf.

Response: Thank you for your comment. We request revisions to the submission form. The statement regarding the funder's role is as follows: "The funders had no role in study design, data collection and analysis, decision to publish, or preparation of the manuscript."

Journal Requirements 3. We note that you have indicated that there are restrictions to data sharing for this study. PLOS only allows data to be available upon request if there are legal or ethical restrictions on sharing data publicly. For more information on unacceptable data access restrictions, please see http://journals.plos.org/plosone/s/data-availability#loc-unacceptable-data-access-restrictions.

Before we proceed with your manuscript, please address the following prompts:

a) If there are ethical or legal restrictions on sharing a de-identified data set, please explain them in detail (e.g., data contain potentially identifying or sensitive patient information, data are owned by a third-party organization, etc.) and who has imposed them (e.g., a Research Ethics Committee or Institutional Review Board, etc.). Please also provide contact information for a data access committee, ethics committee, or other institutional body to which data requests may be sent.

b) If there are no restrictions, please upload the minimal anonymized data set necessary to replicate your study findings to a stable, public repository and provide us with the relevant URLs, DOIs, or accession numbers. For a list of recommended repositories, please see

https://journals.plos.org/plosone/s/recommended-repositories. You also have the option of uploading the data as Supporting Information files, but we would recommend depositing data directly to a data repository if possible.Response: Thank you for your suggestion. We deposit the minimal anonymized dataset and analysis code at GitHub (https://github.com/sociocom/sharedness). Raw text data that might contain personal information cannot be made publicly available under our ethics approval. We therefore release statistically processed data, such as indicator scores, which are sufficient to reproduce all reported results. The dataset reproduces all reported analyses/figures.

- Team-by-week table (SSI, TSI, team-week mean of individual well-being)
- Team-level table (mean IHS per team)
- Emotion table
- Odds ratio table
- All analysis code (versions pinned)

Journal Requirements 4. In the online submission form, you indicated that "The dataset collected and analysed during the current study are not publicly available as the dataset contains sensitive

information regarding personal diaries and individual psychological states, thus precluding its public release but are available from the corresponding author on reasonable request."

All PLOS journals now require all data underlying the findings described in their manuscript to be freely available to other researchers, either 1. In a public repository, 2. Within the manuscript itself, or 3. Uploaded as supplementary information.

This policy applies to all data except where public deposition would breach compliance with the protocol approved by your research ethics board. If your data cannot be made publicly available for ethical or legal reasons (e.g., public availability would compromise patient privacy), please explain your reasons on resubmission and your exemption request will be escalated for approval.

Response: Thank you for your suggestion. As already explained in our response to Journal Requirements 3, we deposit the minimal anonymized dataset and analysis code at GitHub (https://github.com/sociocom/sharedness). Raw text data that might contain personal information cannot be made publicly available under our ethics approval. We therefore release statistically processed data, such as indicator scores, which are sufficient to reproduce all reported results. The dataset reproduces all reported analyses/figures.

- Team-by-week table (SSI, TSI, team-week mean of individual well-being)
- Team-level table (mean IHS per team)
- Emotion table
- Odds ratio table
- All analysis code (versions pinned)

Journal Requirements 5. Thank you for stating the following in the Acknowledgments Section of your manuscript:

“This work was supported by JST-Mirai Program Grant Number JPMJMI21J2, Japan.”

We note that you have provided funding information that is not currently declared in your Funding Statement. However, funding information should not appear in the Acknowledgments section or other areas of your manuscript. We will only publish funding information present in the Funding Statement section of the online submission form.

Please remove any funding-related text from the manuscript and let us know how you would like to update your Funding Statement. Currently, your Funding Statement reads as follows:

“This work was supported by JST-Mirai Program Grant Number JPMJMI21J2, Japan.”

Please include your amended statements within your cover letter; we will change the online submission form on your behalf.

Response: Thank you for your comment. We exclude any mention of funders from the manuscript and request revisions to the submission form. The statement regarding the funder's role is as follows: “The funders had no role in study design, data collection and analysis, decision to publish, or preparation of the manuscript.”

Journal Requirements 6. Please amend either the abstract on the online submission form (via Edit Submission) or the abstract in the manuscript so that they are identical.

Response: We sincerely apologize for the inconsistency in the abstract. We have corrected this issue, and the abstract in the manuscript and the one in the online submission form are now identical.

Journal Requirements 7. Please ensure that you refer to Figure 1 in your text as, if accepted, production will need this reference to link the reader to the figure.

Response: Thank you for pointing this out. We have revised the manuscript to explicitly refer to Figure 1 at its first mention in the text, and we have also placed the figure immediately after that paragraph to facilitate proper linking during production.

Journal Requirements 8. If the reviewer comments include a recommendation to cite specific previously published works, please review and evaluate these publications to determine whether they are relevant and should be cited. There is no requirement to cite these works unless the editor has indicated otherwise.

Response: Thank you for your suggestion. There were no specific recommendations for references from reviewers.

Editor’s Additional Comments

E-1. Title and Abstract Improvements: The term “social harmony” is central to your research but is not clearly reflected in the title or consistently used in the abstract. We suggest rephrasing the title

to better capture this concept, and restructuring the abstract to clearly articulate the study's aim, significance, methodology, and key findings (Reviewer 1 & 2).

Response: Thank you for your comments. We have revised the title to foreground the concept of social harmony and restructured the abstract to more clearly articulate the study's aim, significance, methodology, and key findings.

Title: Social harmony at work: A sharedness index linking team atmosphere to individual well-being in a Japanese company

Abstract: Social harmony, defined as the flourishing of interdependent relationships, is central to well-being in workplace teams but is often measured with abstract self-reports that offer limited interpretability and limited guidance for interventions. This study aims to operationalize social harmony as an interpretable sharedness index and to test its association with individual well-being. In a two-month field study at a Japanese company, 94 employees from 23 teams submitted daily reports containing an 11-point well-being score, an 11-point team atmosphere score, and a short diary entry. We computed two weekly team level indices. The score-based sharedness index was defined as the negative of the within team standard deviation of team atmosphere scores. The text-based sharedness index was defined as the mean semantic similarity among members weekly concatenated diaries using Word Mover's Distance. Across team weeks, score-based sharedness correlated positively with mean individual well-being ($r=0.332$, $p=0.0002$), and text-based sharedness showed a weaker but significant correlation ($r=0.257$, $p=0.003$). Convergent validity with social harmony was indicated by team level associations with the Interdependent Happiness Scale (score-based $r=0.66$, $p=0.019$; text-based $r=0.44$, $p=0.086$). These findings suggest that sharedness reflects a practically interpretable component of social harmony in workplace teams and is positively associated with individual well-being, although causal and intervention effects remain to be tested. The index can be computed from routine ratings and diaries, which supports its use for monitoring and for designing interventions to promote employee well-being.

E-2. Structural and Formatting Revisions: Figures and tables should be referenced explicitly in the text, placed appropriately, and accompanied by concise captions. Currently, many figures (e.g., Fig 1, Fig 2, Fig 4) are inserted mid-paragraph without context, making the flow difficult to follow (Reviewer 1). Reconsider the location of Table 1 and revise Figure 7 caption errors (Reviewer 1).

Response: Thank you for your suggestion. We have repositioned all figures immediately after the paragraph where they are first referenced. As indicated by Reviewer 1, Table 1 is referenced within

the Questionnaire section and has been repositioned immediately after the first relevant paragraph. In addition, we have concisely rewritten the captions for most figures and tables, and we have reviewed and revised the caption for Figure 7.

Figure 1 Caption: Concept overview. The top row shows members' ratings of team atmosphere, while the bottom row presents individual well-being. The left team has inconsistent atmosphere ratings (low sharedness), whereas the right team has consistent ratings (high sharedness). We hypothesize that greater sharedness is associated with higher individual well-being.

Figure 3 Caption: Overview of index construction. (a) SSI: within-team daily SD of atmosphere (sign-reversed), averaged within week. (b) TSI: mean pairwise semantic similarity among weekly concatenated diaries.

Figure 4 Caption: Results for score-based sharedness index. Each plot shows the values per week and per team. Markers of the same color and shape indicate the same team. (a) Result for all teams. The correlation coefficient is 0.332 (p=0.0002), supporting our hypothesis. (b) Results for each team. The "S" indicates the score on the first day, and the "E" indicates the score on the last day. "*" means that the value is statistically significant ($p < 0.1$).

Figure 5 Caption: Results divided by week. "*" means that the value is statistically significant ($p<0.1$). (a) Score-based sharedness index. (b) Text-based sharedness index.

Figure 6 Caption: The correlation coefficient between the sharedness indices and Interdependent Happiness Scale (IHS). Each plot corresponds to each team. Because both correlations exceed 0.4, the indices can serve as surrogate indicators of social harmony.

Figure 7 Caption: Results for text-based sharedness index. Markers of the same color and shape indicate the same team. (a) Result for all teams. (b) Results for each team. The "S" mark indicates the score on the first day and the "E" mark the position of the score on the last day. "*" means that the value is statistically significant ($p<0.1$).

E-3. Editing and Language Usage: Substantive language editing is needed throughout the manuscript. Avoid using ampersands (&) outside of in-text citations and ensure consistency in reference formatting. Reviewer 1 also noted some issues with citation style in the references section, such as the incorrect handling of WHO as an author.

Response: Thank you for your suggestion. We have removed ampersands outside of in-text citations; standardized the reference formatting, and corrected organizational authors entries (e.g., World Health Organization).

Discussion – Principal results

On the other hand, Hareli and Rafaeli [36] proposed a model in which individual mood influences the members of a group, and the cycle of influence continues among group members, causing convergence or divergence of mood. As in Hareli and Rafaeli's model, it is quite possible that individual well-being and sharedness are not simply a relationship in which one affects the other, but are cyclical influences in the interaction among group members. In the future, based on psychological theories such as these, we will use longitudinal data to identify dynamics related to the psychological states of groups and individuals.

Reference

3. World Health Organization. The World Health Report 2001: Mental Health: New Understanding, New Hope. Geneva: World Health Organization; 2001. Available from: https://apps.who.int/iris/handle/10665/42390.

E-4. Missing Standard Sections: Reviewer 2 highlighted missing or underdeveloped sections such as a clear research limitations section, details on ethical considerations, and a discussion on the study's budget or funding. Please expand on these areas to ensure the paper meets standard publication expectations.

Response: Thank you for your suggestion. We have added an *Ethical Considerations* section (covering approval number, informed consent, confidentiality/anonymity, and potential psychological impacts of diaries and feedback). Furthermore, we have reorganized the manuscript by moving the *Limitation*s subsection into a dedicated *Research Limitations* section and clarifying each subtitle. Additionally, we will provide further clarification on funding and the role of the funder in the submission form.

Ethical considerations

This research was approved by the ethics committee of Kyoto University, including Nara Institute of Science and Technology (Review No. 26-P-16), and was conducted in accordance with relevant guidelines and regulations. All participants were fully informed about the study

and provided consent prior to participation. Confidentiality and anonymity were assured, and no personally identifiable or company-confidential information was collected in the diaries. Participation was voluntary, and individuals could withdraw at any time without penalty. Participants and researchers belonged to different organizations, which minimized the risk of coercion or undue influence.

At the same time, ethical attention was required because the daily reporting task itself may have influenced participants' psychological states. Regularly reflecting on one's well-being could heighten self-awareness of mood, which might have both beneficial and burdensome effects. In addition, the feedback system displayed aggregated well-being levels of team members, which could have affected participants' feelings. For example, participants might have experienced distress if others in the team reported low well-being, or reassurance if others reported high well-being. Such potential influences on participants' emotions and team dynamics were carefully considered when designing the study. Future research should further explore ways to minimize unintended psychological impacts while preserving the benefits of self-reflection and collective feedback.

Research Limitations

This study is the first step in one of the few attempts to investigate the dynamics between group psychological states and individual well-being. Thus, there are many challenges ahead of us, as described below.

Correlational Design and Lack of Causality: (…)

Heterogeneity and Potential Mediators Across Teams and Individuals: (…)

Alternative Explanations including Self Report Coupling and Sharedness: (…)

Limited Power in Team and Week Strata (Exploratory $p \leq 0.10$): (…)

Generalizability Limits due to Single Company Sample and Weekly Aggregation: (…)

E-5. Methodological clarity: Enhance the clarity of your methods section by consolidating overlapping content between “Materials” and “Methods,” and introducing a clearly defined “Data Analysis” subsection. Reviewer 2 also requested that you elaborate more on sample size, population details, and statistical methods used.

Response: Thank you for your suggestion. We have merged the overlapping content into a single *Materials and Methods* section and added a clearly defined *Data Analysis* subsection. This subsection includes a subsubsection on *Analytical framework*, which provides clarifications on the population, inclusion and exclusion criteria, weekly window, and the definitions of SSI and TSI. We have also added a subsection on *Statistical analysis* for clarifying the statistical procedure (two-sided Pearson correlation). Furthermore, additional demographic information such as participants' age and gender has been added to the *Participants* subsection.

Materials and Methods

Participants: (…) Each team comprised four to seven members, and the number of members who submitted one or more daily reports ranged from one to seven per team. The average age of participants was 39.4 years, and 27.5% were female. (…)

Participant consent: (…)

Questionnaire: (…)

Instructions: (…)

Data Analysis: (…)

Analytical framework: The analytical unit was the team by week. Weeks were defined as consecutive seven-day periods anchored at 2022-09-01; Week 1 began on 2022-09-01 and covered the period from 2022-09-01 to 2022-09-07. Aggregations honored these fixed seven-day windows. For each team and week, we aggregated daily inputs to weekly summaries and constructed two team-level sharedness indices (score-based and text-based) that operationalize social harmony. We then examined their associations with the weekly mean of individual well-being across team members. An overview of the workflow is shown in Fig 3. This was a non-interventional observational study with daily self-reports over nine weeks (from 2022-09-01 to 2022-10-31). Team-level sharedness indices (SSI, TSI) were computed weekly, and the Interdependent Happiness Scale (IHS) was administered once after the reporting period. No randomization or experimental manipulation was performed.

Score-based sharedness index: (…)

Text-based sharedness index: (…)

Linguistic analysis: words and emotions: (…)

Statistical analysis: Unless noted otherwise, the procedures below apply to both sharedness indices (SSI and TSI). We computed two-sided Pearson correlation coefficients between each sharedness index (SSI, TSI) and the weekly mean of individual well-being across all team by weeks, with $\alpha = 0.05$. We report Pearson correlations r with two-sided p values. We then repeated the correlations within teams and within weeks as exploratory analyses; owing to smaller sample sizes, we treat p 0.10 as suggestive evidence and report unadjusted p-values. Exclusions and aggregation rules followed the criteria described above. Analyses were conducted in Python 3.10.18 (pandas 2.3.1, scipy 1.15.3); exact versions and executable scripts are available in the project repository (see Data Availability Statement)

Reviewer #1

R1-1. I suggest re-phrasing the title to reflect the concept of ‘social harmony’ since it is the focus in the abstract. And also refer to it in the body of the paper.

Response: Thank you for your suggestion. We have revised the title to foreground the concept of social harmony. The specific changes are described in our response to Comment E-1.

R1-2. Editing is required.

Response: Thank you for your comment. We have standardized the style, and the specific changes are described in our response to Comment E-2.

R1-3. The abstract needs further improvement since the study aim, gap, significance are not clearly introduced. Besides, the model of analysis should be documented.

Response: We appreciate the reviewer’s suggestion. We have restructured the abstract to include the study aim, significance, methods, key findings, and conclusion. We have also added

a succinct description of the analysis model. Details of these revisions are provided in our response to Comment E-1.

R1-4. Figure 1 appears all of a sudden when the paragraph hasn't come to an end. Besides, there is no reference to it, before or after.

Response: Thank you for your suggestion. We have moved Figure 1 to follow its first mention. The specific changes are described in our response to Comment E-2.

R1-5. Table one appears in the middle of the paragraph on 'Participant' with no reference to it after or before it. It is not in the right place. It should come as part of the Questionnaire section.

Response: Thank you for your comment. We have moved Table 1 to the *Questionnaire* subsection and referenced it there. The specific changes are described in our response to Comment E-2.

R1-6. Figure 2 should be situated after the first paragraph of the Instructions section.

Response: Thank you for your suggestion. We have repositioned all figures immediately after the paragraph where they are first referenced. The specific changes are described in our response to Comment E-2.

R1-7. It is better to merge both sections Materials and Methods since both deal with the methodological consideration or the Methodology of the paper.

Response: Thank you for your comment. We have merged the *Materials* and *Methods* sections into a single *Materials and Methods* section. The specific changes are described in our response to Comment E-5.

R1-8. The Analysis part of the Methods section should be separated and titled 'Analysis' or 'Data Analysis'.

Response: Thank you for your suggestion. We have added a dedicated *Data Analysis* subsection in the *Materials and Methods* section. The specific changes are described in our response to Comment E-5.

R1-9. No reference to Figure 3 is seen in the main text.

Response: Thank you for your suggestion. We inserted an in-text reference immediately before Figure 3.

R1-10. I suggest having an introductory statement or account before Figure 4 to prepare the reader to what comes after. Besides, the caption of the figure is too long. All figures are followed by very long captions. Reduce, please.

Response: Thank you for your suggestion. Figure 4 has been moved immediately after the paragraph with the related description; captions across figures have been shortened. The specific changes are described in our response to Comment E-2.

R1-11. When explaining in the section 'text-based sharedness index', in the second paragraph, Fig 7 is mentioned while this section is followed by Fig. 6. Mismatch is there in different places.

Response: Thank you for your suggestion. We have repositioned Figure 7, which shows the results of the "text-based sharedness index," immediately after the first paragraph of the *Text-based sharedness index* section, in accordance with the content.

R1-12. The ampersand '&' should not be used within the text (as in line 372 and some other places), only used within the 'in-text citation'.

Response: Thank you for pointing it out. We have removed ampersands outside citations. The specific changes are described in our response to Comment E-3.

R1-13. In the third reference in the Reference list, spell the whole words of WHO rather than treating the title of the organization as an authors' name.

Response: Thank you for your comment. We have corrected to "World Health Organization" and modified other organizational authors. The specific changes are described in our response to Comment E-3.

R1-14. References in the Reference List need double checking for some minor issues.

Response: Thank you for your suggestion. We carefully reviewed the reference list and corrected several entries that did not conform to the PLOS ONE formatting requirements.

Reviewer #2

R2-1. In a structured abstract, it can include a statement that states the main purpose of the research. It should be included only in the introduction of topics that do not require references. Method: It should be stated precisely, including the study population, sample size, analysis methods, etc. The findings should be stated by stating the most important topic and based on statistical analysis, the topics should be raised. If each hypothesis is meaningful, state it by stating its significance. Conclusion: It should be expressed based on the study findings with a more general approach.

Response: Thank you for your comment. We fully restructured the *abstract* to clearly articulate the study's aim, significance, methodology, and key findings. The specific changes are described in our response to Comment E-1.

R2-2. Introduction: Adhere to a more precise structure. By stating the importance of the topic and addressing it in the world and the Asian region, address the growing or declining trend of this topic and by examining this topic and defining the relationship between variables and its impact role in Japanese companies and comparing it with a systematic approach, discuss the rationale and explanation of the why and philosophy of your research. The article that is used as a research method should be included in the method and methods section.

Response: We appreciate the reviewer's constructive feedback. We have reorganized the *Introduction* to address the global and Asian policy context, outline theoretical relationships, and present the rationale for focusing on the Japanese corporate setting. We also explain how our sharedness indices complement standardized scales (e.g., IHS, Harmony in Life). Furthermore, methodological sources used in our approach (e.g., WMD, tokenization/embeddings) have been appropriately cited in the *Materials and Methods* section.

Introduction

Well-being encompasses various aspects of life, including the development of one's potential, personal agency, sense of purpose, and positive relationships [1]. It is often considered a more reliable social success than statistical indices, such as economic indices [2]. The World Health Organization (WHO) defines it as being closely associated with positive mental health [3], and higher well-being has been associated

with favorable outcomes in physical health and longevity [4], performance at work [5], and economic wealth [6, 7].

In parallel with this scholarly interest, policy and standards have shifted toward workplace well-being over roughly the past decade. National measurement frameworks moved beyond GDP, for example the OECD's How's Life? indicators that mainstreamed subjective well-being in official statistics [8]. Organizational guidance has expanded, with ISO 45003 published in 2021 on managing psychosocial risks [9] and the WHO and the International Labour Organization (ILO) issuing joint guidelines on mental health at work in 2022 [10]. In Asia, policy uptake is visible. Japan's national Stress Check System was introduced in response to growing concerns about work-related mental disorders and karo-shi (death from overwork). Since 2015, the system has legally required workplaces with 50 or more employees to conduct annual screening of psychosocial stressors. The program is intended not only to provide workers with individual feedback but also to generate workplace-level data that can guide organizational improvements [11, 12]. Singapore's Tripartite Advisory on Mental Well-Being at Workplaces emerged from concerns about sustaining productivity and inclusiveness in the context of changing work arrangements, such as remote work, and an ageing workforce. It was jointly issued by government, employers, and unions, and it promotes organization-level practices including flexible work arrangements, anti-stigma policies, and peer support systems, all designed to safeguard psychological health and foster social integration at work [13]. Taken together, these developments document a sustained, and still expanding, policy and practice emphasis on workplace well-being in both global and Asian contexts.

A long-standing perspective distinguishes affective and cognitive components of well-being. On the affective side, a higher frequency of positive emotions and a lower frequency of negative emotions are thought to contribute to well-being; the Positive and Negative Affect Schedule (PANAS) is a widely used instrument to assess this dimension [14]. On the cognitive side, two constructs are often highlighted: life satisfaction, defined as an overall evaluation of one's life according to self-selected criteria [15], and social harmony, referring to the flourishing of interdependent relationships within a social context [16]. Instruments such as the Interdependent Happiness Scale [17] and the Harmony in Life Scale [18] have extended this cognitive tradition by emphasizing the social harmony dimension that is particularly relevant in interdependent settings.

In workplaces, well-being is linked to outcomes of clear practical importance, including performance, productivity, and retention [5]. While a substantial body of research has examined affective experiences at work and life satisfaction in occupational contexts, social harmony within work teams remains comparatively underexplored. Existing social harmony scales are valuable for broad assessment, yet their items are relatively abstract, which can make interpretation and day-to-day intervention at the team level challenging. There is a need for indicators that preserve conceptual alignment with social harmony while improving interpretability and actionability in organizational practice.

Building on shared reality theory [19, 20], we conceptualize sharedness as the degree to which team members converge in their assessment of the team's overall psychological state. In our framework, sharedness serves as a concrete, team-level operationalization of social harmony. We emphasize that sharedness does not imply that members have similar levels of individual well-being; rather, it reflects similarity in members' evaluations of the team atmosphere as a whole, that is, a shared appraisal of how the team is doing overall on a given day (Fig 1). Conceptually, higher sharedness may reduce ambiguity about the team's situation, facilitate coordination of expectations, and support prosocial responses among coworkers, which in turn could relate to higher individual well-being. This rationale is especially pertinent in Japanese companies, where interdependent coordination and consensus-oriented practices can make a shared understanding of the team's state particularly consequential for day-to-day functioning.

Among various possible team psychological states related to sharedness, this study employs the following two different indices:

- Score-based sharedness: The agreement of team atmosphere score
- Text-based sharedness: The agreement of diary contents

For qualitative sharedness, the diary text was analysed using Natural Language Processing (NLP). This qualitative index has the advantage of flexibility, as it can be calculated using only textual data, allowing for more nuanced insights into team dynamics without the need for extensive quantitative measures. These indices are designed to be computable from routinely collected ratings and diaries, easy to

interpret, and amenable to intervention in the workplace compared to traditional measures.

Within this framework, we examine the relationships among sharedness, social harmony, and individual well-being in Japanese work teams. We state three hypotheses. First, higher score-based sharedness will be positively associated with the weekly mean of individual well-being across team members. Second, higher text-based sharedness will likewise be positively associated with the weekly mean of individual well-being. Third, both indices will show positive correlations with an established social harmony measure at the team level (Interdependent Happiness Scale). These expectations are grounded in the interdependent coordination that characterizes many Japanese companies, where shared understanding is often integral to effective collaboration.

In positioning this study, we explicitly compare our approach with systematic, survey-based approaches that are widely used to assess well-being and social harmony (e.g., the Interdependent Happiness Scale and Harmony in Life). Systematic approaches rely on standardized items administered at planned intervals and offer strong psychometric grounding and comparability across groups. By contrast, our sharedness indices are designed to be team-anchored and high-frequency: the score-based index captures within-team agreement in atmosphere ratings, and the text-based index captures semantic convergence in routine diaries. This design aims to complement standardized scales in four ways: (i) unit of analysis (team-level convergence rather than only individual attitudes), (ii) temporal granularity (weekly summaries from daily inputs rather than infrequent cross-sections), (iii) interpretability for interventions (direct signals of team consensus or fragmentation), and (iv) respondent burden (using existing diaries with minimal additional load). We do not seek to replace validated instruments; instead, we assess convergent validity by relating our indices to IHS at the team level and examine their association with weekly mean individual well-being in Japanese work teams. Operational definitions and analytic procedures are detailed in the Materials and Data Analysis sections. Our rationale is pragmatic: to preserve the conceptual core of social harmony while offering an interpretable, low-burden, team-level indicator that can inform timely workplace actions.

Data Analysis – Text-based sharedness index

(...) Semantic distances were computed using Word Mover's Distance (WMD) [22] with the Japanese Wikipedia Entity Vectors pre-trained Word2Vec model (300-dimensional; jawiki.all vectors.300d.txt.bz2) [23, 24]. Tokenization for Japanese texts followed MeCab [25] using the NEologd [26] dictionary.

R2-3. Method: By designing a model of the whole except in detail about the implementation method, statistical population and formula in a title, how to collect data, tools or data on official sites, how to process and finally from statistical software and methods.

Response: Thank you for your suggestion. In the *Data Analysis* subsection, we have added a subsubsection on *Analytical framework* for clarify the population, inclusion/exclusion, weekly window, and SSI/TSI definitions. We have also added a subsection *Statistical analysis* to describe the statistical procedure (two-sided Pearson correlation). In addition, information regarding the participants' age and gender has been added to the *Participants* subsection. Details of these revisions are provided in our response to Comment E-5.

R2-4. All the results of data analysis in the findings section should be presented in tables starting with the most important findings for better summary and understanding.

Response: Thank you for your suggestion. We have integrated the main results into a single summary table (Table 2, placed immediately after the second paragraph of the *Results* section) and added an additional table for the results on *Emotions* (Table 4).

Results

Table 2. Primary associations of sharedness indices with individual well-being and IHS. Pearson correlations (two-sided). For WB, N is the number of team by weeks; for IHS, N is the number of teams. Abbreviations: WB, individual well-being; IHS, Interdependent Happiness Scale; SSI, score-based sharedness index; TSI, text-based sharedness index.

	WB (team by week)			IHS (team level)		
	r	p	N	r	p	N
Score-based sharedness index	0.332	0.0002	118	0.662	0.019	12
Text-based sharedness index	0.257	0.0030	133	0.443	0.086	16

Results - Linguistic analysis: words and emotions – Emotions

Table 4. Individual well-being by predicted emotion (emotion labels are outputs of the LIFE STORY–trained classifier). Δ = WB_emotion – WB_all; positive values indicate higher well-being than the overall mean, negative values indicate lower.

Emotion	*N*	WB Mean	WB SD	Δ vs. overall mean
All data	1741	6.800	2.140	–
Joy	647	7.540	1.750	0.730
Trust	498	7.290	1.690	0.490
Anxiety	302	5.490	2.200	-1.310
Surprise	103	6.670	2.320	-0.130
Sadness	72	5.780	2.450	-1.020
Anger	63	4.980	2.530	-1.830
Disgust	56	4.680	2.120	-2.120

R2-5. Regarding ethics in research in this research, it should be mentioned

Response: Thank you for your suggestion. We have added the *Ethical Considerations* section that specifies the approval number, informed consent, confidentiality/anonymity, and potential psychological impacts of diaries and feedback. The specific changes are described in our response to Comment E-4.

R2-6. In the area of budget of this research, it should be mentioned

Response: Thank you for your suggestion. We have removed any mention of funders from the manuscript and requested the necessary revisions to the submission form. As stated in the cover letter, the funder's role is disclosed as follows: “The funders had no role in study design, data collection and analysis, decision to publish, or preparation of the manuscript.” Please also see our comments and responses for Journal Requirements 5.

R2-7. Research limitations should be mentioned in detail as a title: In general, the precise and intelligent title of this article can help with the mentioned amendments in a more accurate presentation of those interested in this topic

Response: Thank you for your suggestion. We have added a section of *Ethical Considerations* and moved the *Limitations* subsection to the *Research Limitations* section and clarified each subtitle. The specific changes are described in our response to Comment E-4.
