# Supplementary material for: Social harmony at work: A sharedness index linking team atmosphere to individual well-being in a Japanese company
Source: PLoS One. 2025 Dec 29;20(12):e0336368. doi: 10.1371/journal.pone.0336368 (PMC12747401; doi:10.1371/journal.pone.0336368)
Supplement: Attachment — Submitted filename: Response_to_Reviewers_auresp_2.pdf [file pone.0336368.s006.pdf]

Thank you very much for your email dated December 1, 2025, and for the provisional acceptance of our manuscript. We are delighted to hear that the reviewers are satisfied with our revisions.

We have addressed the minor corrections pointed out by Reviewer #4 regarding the consistency of statistical symbols. We have carefully reviewed the manuscript and made the necessary corrections.

Our point-by-point responses to the specific comments are listed below.

[Reviewer #4 Comment 1] line 274: there is a missing symbol to indicate < for the p-value, and also in the caption for Figure 4 the symbol is not correct.

Response: Thank you for pointing out these errors. We have corrected the missing symbol in line 274 and the incorrect symbol in the caption for Figure 4.

Line 274: (...) to smaller sample sizes, we treat $p < 0.10$ as suggestive evidence and report

Figure 4 – Caption: Results for score-based sharedness index. Each plot shows the values per week and per team. Markers of the same color and shape indicate the same team. (a) Result for all teams. The correlation coefficient is 0.332 (p=0.0002), supporting our hypothesis. (b) Results for each team. The “S” indicates the score on the first day, and the “E” indicates the score on the last day. “*” means that the value is statistically significant ($p < 0.1$).

[Reviewer #4 Comment 2] lines 305 and 328 have <= and the captions for Fig 5 and 7 have < alone - this should be consistent.

Response: We apologize for these multiple errors. We have corrected lines 305 and 328 to use the "<" symbol, making them consistent with the captions for Figures 5 and 7. Additionally, we identified and corrected a similar instance in line 488 to ensure full consistency throughout the manuscript.

Line 305: (...) showed significantly positive correlations at $p < 0.10$: (Weeks 3, 4, 7, 8, (...)

Line 328: (...) significantly positive correlations at $p < 0.10$:(Weeks 3, 4, 6, 7, and 8), (...)

Line 488: Limited Power in Team and Week Strata (Exploratory $p < 0.10$)
